# Psychiatric disorder associated with vacuum-assisted breast biopsy clip placement: a case report

**DOI:** 10.1186/1752-1947-2-332

**Published:** 2008-10-17

**Authors:** George C Zografos, Flora Zagouri, Theodoros N Sergentanis, Marios Panou, Dimitrios Dardamanis, Georgia Giannakopoulou, George M Filippakis, George Papadimitriou, Sophia Stamouli

**Affiliations:** 1 Breast Unit, 1st Department of Propaedeutic Surgery, Hippokratio Hospital, School of Medicine, University of Athens, Vas Sofias Avenue, Athens 116 27, Greece; 2 Department of Psychiatry, Eginition Hospital, University of Athens, Greece

## Abstract

**Introduction:**

Vacuum-assisted breast biopsy is a minimally invasive technique that has been used increasingly in the treatment of mammographically detected, non-palpable breast lesions. Clip placement at the biopsy site is standard practice after vacuum-assisted breast biopsy.

**Case presentation:**

We present the case of a 62-year-old woman with suspicious microcalcifications in her left breast. The patient was informed about vacuum-assisted breast biopsy, including clip placement. During the course of taking the patient's history, she communicated excellently, her demeanor was normal, she disclosed no intake of psychiatric medication and had not been diagnosed with any psychiatric disorders. Subsequently, the patient underwent vacuum-assisted breast biopsy (11 G) under local anesthesia. A clip was placed at the biopsy site. The pathological diagnosis was of sclerosing adenosis. At the 6-month mammographic follow-up, the radiologist mentioned the existence of the metallic clip in her breast. Subsequently, the woman presented complaining about "being spied [upon] by an implanted clip in [her] breast" and repeatedly requested the removal of the clip. The patient was referred to the specialized psychiatrist of our breast unit for evaluation. The Mental State Examination found that systematized paranoid ideas of persecutory type dominated her daily routines. At the time, she believed that the implanted clip was one of several pieces of equipment being used to keep her under surveillance, the other equipment being her telephone, cameras and television. Quite surprisingly, she had never had a consultation with a mental health professional. The patient appeared depressed and her insight into her condition was impaired. The prevalent diagnosis was schizotypal disorder, whereas the differential diagnosis comprised delusional disorder of persecutory type, affective disorder with psychotic features or comorbid delusional disorder with major depression.

**Conclusion:**

This is the first report of a psychiatric disorder being brought to the fore using a vacuum-assisted breast biopsy clip. Vacuum-assisted breast biopsy, and breast biopsy in general, represent a significant experience, encompassing anxiety and pain; it may thus aggravate psychiatric conditions. Apart from these well-established factors, other aspects, such as the clip, may occasionally become significant. In a modern breast unit, the evaluation of patients should be multidisciplinary. A psychiatrist may be needed for optimal management of anxiety-related issues, as well as for the detection of psychiatric disorders.

## Introduction

Vacuum-assisted breast biopsy (VABB) is a minimally invasive technique increasingly use in the diagnosis of mammographically detected, non-palpable breast lesions [1,2]. VABB is effective on lesions both with and without microcalcifications [3,4].

It is standard practice to place a clip at the biopsy site at the end of the procedure [5] in order to locate the biopsy site in the case of subsequent malignancy. To date, the only clip-related complications in the literature concern clip migration [6,7].

This is the first report of an unusual sequela after clip placement: the unexpected occurrence of a psychiatric incident.

## Case presentation

We present the case of a 62-year-old Caucasian woman who is divorced and lives alone. Suspicious microcalcifications (BI-RADS 4A) were detected in her left breast. Subsequently, VABB was scheduled; the patient was informed about the procedure, including the clip placement, by the surgeon performing the intervention. During the course of taking the patient's history, she communicated excellently, her demeanor was normal, she disclosed no intake of psychiatric medication and had not been diagnosed with any psychiatric disorders. According to the standard practice of the unit, the patient gave her written informed consent without any problem.

Subsequently, the patient underwent VABB (Mammotome; Ethicon Endo-Surgery Inc, Johnson & Johnson, Cincinnati, OH, USA). VABB was performed on a digital prone table (Mammotest, Fischer Imaging, Denver, CO, USA) using 11-gauge vacuum probes, under local anesthesia. The examination proceeded according to a standard protocol to ensure quality control. A radiologist was present to assist in the targeting. A mammogram of the specimens taken after VABB confirmed the excision of microcalcifications. A clip was placed at the biopsy site. The pathological diagnosis was sclerosing adenosis.

At the 6-month mammographic follow-up, the radiologist mentioned the existence of the metallic clip in her breast. Subsequently, the woman presented to our breast unit complaining about "being spied [upon] by an implanted clip in [her] breast" and repeatedly requested the removal of the clip.

From the interaction with the nursing and medical staff, it was apparent that she was irritable, dysphoric, very suspicious and verbally aggressive. The patient was referred to the specialized psychiatrist of our breast unit for evaluation.

The Mental State Examination found the existence of systematized paranoid ideation of persecutory type dominating the daily routines of the patient. At the time, she believed that the implanted clip was one of several pieces of equipment being used to keep her under surveillance.

During the psychiatric interview, the patient revealed symptoms originating 11 years previously, at the time of her divorce, when she felt that her ex-husband was paying some religious organizations to spy on her, especially when she was at home. She stated that this was being done through the telephone, cameras, television and other technologically advanced equipment using electromagnetic waves. The patient had never consulted a mental health professional.

The thought content of the patient was also positive for ideas of reference and her speech was of normal rhythm, rate and quantity.

The patient appeared depressed, but occasionally she expressed anger about what was happening to her and she reported anhedonia, helplessness, hopelessness and decreased concentration. She had no suicidal thoughts.

As far as her social life was concerned, she reported having gradually decreased her social contacts over the past 10 years, as she believed that all of her friends were under surveillance as well and that this seriously disrupted their lives. The patient did not have any disturbances in perception, or in her thought processes. She did not have any insight into her psychiatric condition.

It should also be kept in mind that the patient has never met criterion A of schizophrenia. In conclusion, with respect to chronicity, the paranoid ideation first appeared approximately 10 years ago and since then has been chronic.

The prevalent diagnosis was schizotypal disorder encompassing paranoid or bizarre ideas not amounting to true delusions, anhedonia, disaffection and a tendency toward social withdrawal; the differential diagnosis comprised delusional disorder of persecutory type, affective disorder with psychotic features or delusional disorder and comorbid major depression.

## Discussion

This is the first report of a psychiatric disorder being brought to the fore from the use of a VABB clip. Indeed, this is a rather rare case: more than 800 VABB procedures have been performed in our breast unit and no comparable cases have been noted.

VABB represents a significant experience. Its psychological impact on the patient is noteworthy, as documented at a psychoendocrinological level [8]. In addition, pain in VABB is a major parameter, which merits study and evaluation [9]. Fear and anxiety before the biopsy, the biopsy procedure *per se*, and the pain experienced all represent multiple contributory factors exerting significant psychological pressure upon the patient, irrespective of the outcome. Unexpected aggravating factors, such as the clip, might also be present. Thus, exacerbation of underlying psychiatric disorders, after either a malignant or a benign finding, may be expected; this possibility should not be underestimated. Unfortunately, the literature has not shed light upon this subject.

Nevertheless, for the optimal interpretation of the above, some limitations have to be addressed. Given the presence of ideation in the past, the emergence of paranoid ideas may not be completely unexpected. In addition, in this patient, the clip seemed to play the major triggering role; in a broader context, however, other parts of the breast biopsy-related experience may similarly be proven important. At any rate, the nature of this case report is one of observation and suggestion; in the absence of systematic studies assessing the incidence of triggered psychiatric conditions, caution is warranted in drawing conclusions about the possibility of breast biopsies exacerbating psychiatric conditions or bringing them to the fore.

All in all, it seems quite surprising that a breast-related procedure was the stimulus for the diagnosis of the psychiatric disorder. The evaluation of patients in a modern breast unit should be multidisciplinary. A psychiatrist may be required for the optimal management of anxiety-related issues as well as for the detection of psychiatric disorders.

## Conclusion

This is the first report of a psychiatric disorder being brought to the fore as a result of the use of a VABB clip. VABB, and breast biopsy in general, represents a significant experience, encompassing anxiety and pain; it may thus aggravate psychiatric conditions. Apart from these well-established factors, other aspects, such as the clip, may occasionally become significant. In a modern breast unit, the evaluation of patients should be multidisciplinary. A psychiatrist may be required for the optimal management of anxiety-related issues, as well as for the detection of psychiatric disorders.

## Abbreviations

VABB: vacuum-assisted breast biopsy

## Competing interests

The authors declare that they have no competing interests.

## Authors' contributions

GCZ supervised and performed the VABB, critically revised the manuscript for important intellectual content and gave final approval of the version to be published. FZ wrote the manuscript and reviewed the international literature. TNS wrote the manuscript and interpreted the case findings with respect to the international literature. MP participated in the interpretation of the psychiatric findings and contributed to the review of the literature. DD performed the VABB. GG made the radiological evaluation and assisted in the VABB; both DD and GG helped in editing the manuscript. GMF performed the VABB and participated in the evaluation of the case findings. GP revised the manuscript for important intellectual content and contributed to the psychiatric evaluation. SS performed the Mental State Examination and revised the manuscript for important intellectual content.

## Consent

Written informed consent was obtained from the patient and her next-of-kin for publication of this case report and any accompanying images. A copy of the written consent is available for review by the Editor-in-Chief of this journal.
